# Esthetic rehabilitation of the smile with partial laminate veneers in an older adult

**DOI:** 10.1002/ccr3.1593

**Published:** 2018-06-04

**Authors:** Romain Ceinos, Valérie Pouyssegur, Yves Allard, Marie‐France Bertrand

**Affiliations:** ^1^ Department of Conservative Dentistry and Endodontics UFR Odontologie Université Côte d’Azur 24 Avenue Diables Bleus 06357 Nice cedex 4 France; ^2^ Department of Prosthodontics UFR Odontologie Université Côte d’Azur 24 Avenue Diables Bleus 06357 Nice cedex 4 France; ^3^ MICORALIS Université Côte d’Azur 24 Avenue Diables Bleus 06357 Nice cedex 4 France; ^4^ Present address: UFR Odontologie Université Côte d’Azur Nice France; ^5^ Present address: MICORALIS Université Côte d’Azur Nice France

**Keywords:** conservative dentistry, elderly, esthetic, partial veneer

## Abstract

Bonded partial ceramic veneer is an interesting alternative to full buccal restoration in terms of tissue economy. However, its usage is restricted to patients with a low smile line (such as the elderly) due to the visibility of the tooth/restoration limit which is a key to the therapeutic decision.

## 1 INTRODUCTION

As life expectancy increases, more and more elderly people are concerned with their appearance, and a beautiful and esthetic smile is of the essence.^1^ However, age causes morphological changes (such as a lower smile…) which may require specific treatment and/or allow alternative procedures. New procedures, materials, and dentistry techniques provide senior people with an improved quality of life and a greater self esteem^2^ due to a confident esthetic smile.

## 2 CASE HISTORY

A 78‐year‐old woman presented herself for a consultation after a fall that caused the fracture of her two maxillary incisors.

**Figure 1:**
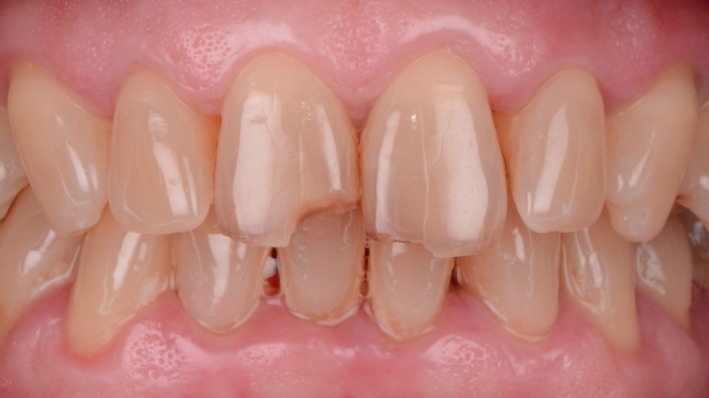
Initial situation (Intraoral view): The left incisor has a large mobile enamel chip and the right incisor has a fracture of the mesial angle without pulp exposure

A clinical examination revealed no injury to the cutaneous teguments and asymptomatic teeth. The teeth of the anterior maxillary block were healthy and positively responded to a pulp sensitivity test (a frozen cotton pellet was placed in the cervical area of the teeth). The traumatic fractures resulted in the loss of fragments on the incisal edges with no pulp exposure (enamel‐dentin fracture) on the right maxillary incisor and of a voluminous enamel chip (enamel fracture) on the left maxillary incisor extending to the buccal upper third of the crown (Figure 1).

During the interview, the patient complained of disfigurement and reported that she was self‐conscious because of her injury. Furthermore, the patient stated that she did not like the shape of her incisors and that there was “too much space between adjacent teeth.”

During the esthetic interview, the patient presented a low smile line in the maximum phase (low‐type smile) revealing only half the buccal surfaces of her teeth without the appearance of the gingiva.

The clinical case presented here is consistent with the observations of Wulfman et al who recently reported that the elderly show an increasing interest for their dental esthetic, especially in women who are twice as willing as men to alter their smile.^3^ Interestingly, color change seems not to be the primary concern for this population.^4^, ^5^

## 3 INVESTIGATIONS AND TREATMENT

While considering the primary objective which was to provide the patient with the highest possible esthetic result, all currently available treatments were considered for this case: ceramic or composite, direct or indirect veneers, partial or full buccal restoration.

Although ceramic is more prone to microfracture and has a lower elasticity coefficient than composite resin, it still provides the highest esthetic results.

Indirect veneers allow a good color stability and a high resistance to saliva, yet they are still very costly and time‐consuming as they require many preliminary steps (wax up, adaptations…). Direct veneers are cheaper and quicker to use from a practitioner point of view, and although they require a high level of skill, they still allow the obtaining of an interesting esthetic result.

When compared to full buccal restoration, partial veneers allow the preservation of sound enamel for tissue economy, ensures periodontal health and provides high esthetical results^6^. However, the limit of the restoration may be visible with an impact on the esthetic outcome.

Because of the high esthetical demand of the patient, and due to the volume of the dental substance loss, to the enamel mobile fragments (in which the cracks continued to the midheight of the crown), and to the low position of the smile (which would conceal the restoration’s edge), direct partial ceramic veneers were selected for the treatment of this case.

The patient was then informed about the intended treatment and her consent was obtained (photograph consent form validated by the Quality Commission of the Pole of Odontology of the University Hospital of Nice).

The objective of this study was to demonstrate that a high esthetic demand of an elderly patient can be met using modern partial bonded ceramic techniques.

## 4 PROCEDURAL STEPS

The treatment was conducted as follows:

1. The initial dental color was recorded in a humid environment under ideal light conditions (natural daylight 6500°K, Gamain^®^ brand lighting, Tauxigny, France). The selected color was 5M2 (3D‐Master Vita, Vita Zahnfabrik, Bad Säckingen, Germany) (Figure 2).

**Figure 2:**
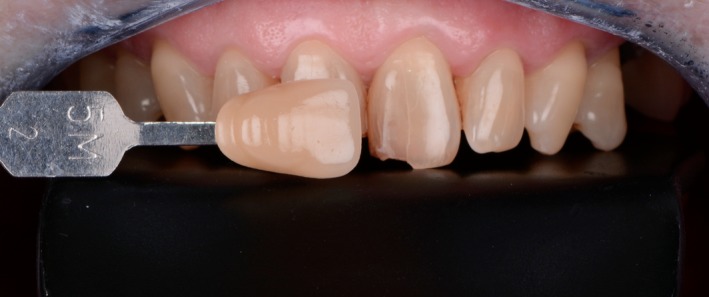
Color selection
2. After removing the mobile enamel fragment, the dental surfaces were prepared using calibrated burs for the teeth that were visible during the maximum smile phase (Figure 3).

**Figure 3:**
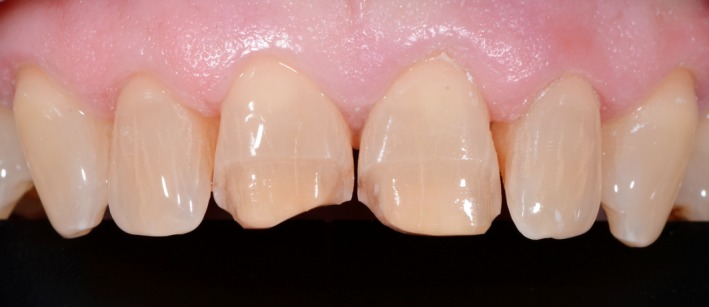
Dental tissues after the minimum preparation for partial veneers (the goal of the preparation was to retain the maximum amount of enamel)
3. The double silicone (Hydrorise Putty and Light, Zhermack^®^, Badia Polesine, Italy) impression provided a working model with preparation limits that were very easily achieved at the midheight of the crown (Figure 4). Two partial ceramic (using Lithium Disilicate glass‐ceramic) veneers with contours that met the patient’s shape requirements were constructed.

**Figure 4:**
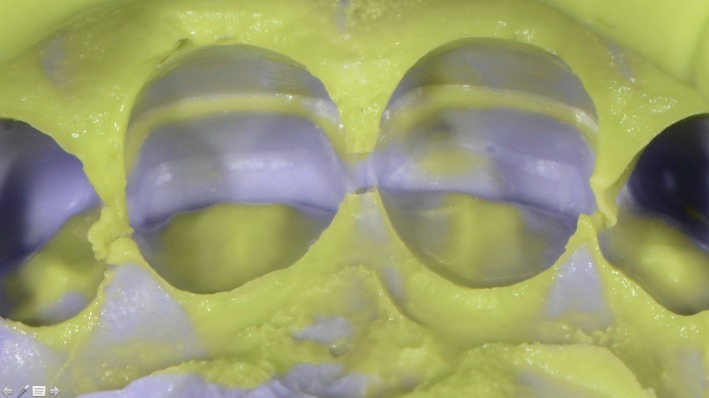
Double silicone impression with high magnification of the preparation limits
4. After fitting, the intrados of the veneers were initially conditioned by acid etching with 9.5% fluoric acid (Ultradent^®^Porcelain Etch, Ultradent^®^, Utah, United States) for 30 seconds, then rinsed with water before being exposed to 35% orthophosphoric acid (Ultra‐Etch^®^etchant, Ultradend^®^) for 20 seconds (to eliminate the creation of mineral salts), and finally abundantly rinsed with water. Once the intrados was dry, a silane (Ultradent^®^Porcelain Silane, Ultradent^®^) was applied, and the partial veneers were allowed to stand for one minute (Figure 5).

**Figure 5:**
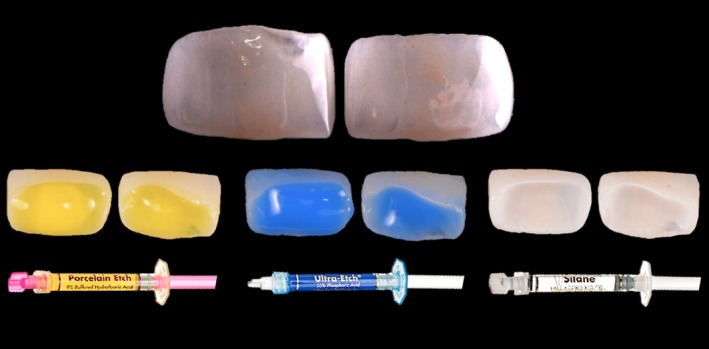
Conditioning of the intrados of the partial veneers
5. The teeth were isolated from moisture using a rubber dam (Nic Tone^®^ Hard, MdC Dental^®^, Gardenna, United States). The dental tissues were conditioned to meet the bonding requirements (the adjacent teeth were protected by a metal matrix strip). After differential etching of the enamel and dentin surfaces (10 seconds for dentin at the fracture level and 30 seconds for enamel) with a 35% phosphoric acid solution (Ultra‐Etch^®^etchant), the teeth were rinsed with water for 40 seconds and then dried with a #3 cotton pellet (Richmond Dental & Medical, Charlotte, United States) to keep the dentin surfaces slightly moist. The bonding agent (Peak^®^Universal Bond, Ultradent^®^) was applied with a scrubbing motion for 10 seconds (to ensure that the resin deeply penetrated into the dentinal tubules on the edge of the fracture area); then, the veneers were dried for 10 seconds using gentle air blown on all surfaces and exposed to light curing for 10 seconds (Bluephase^®^ Style LED, Ivoclar‐Vivadent^®^, Schaan, Liechtenstein).

**Figure 6:**
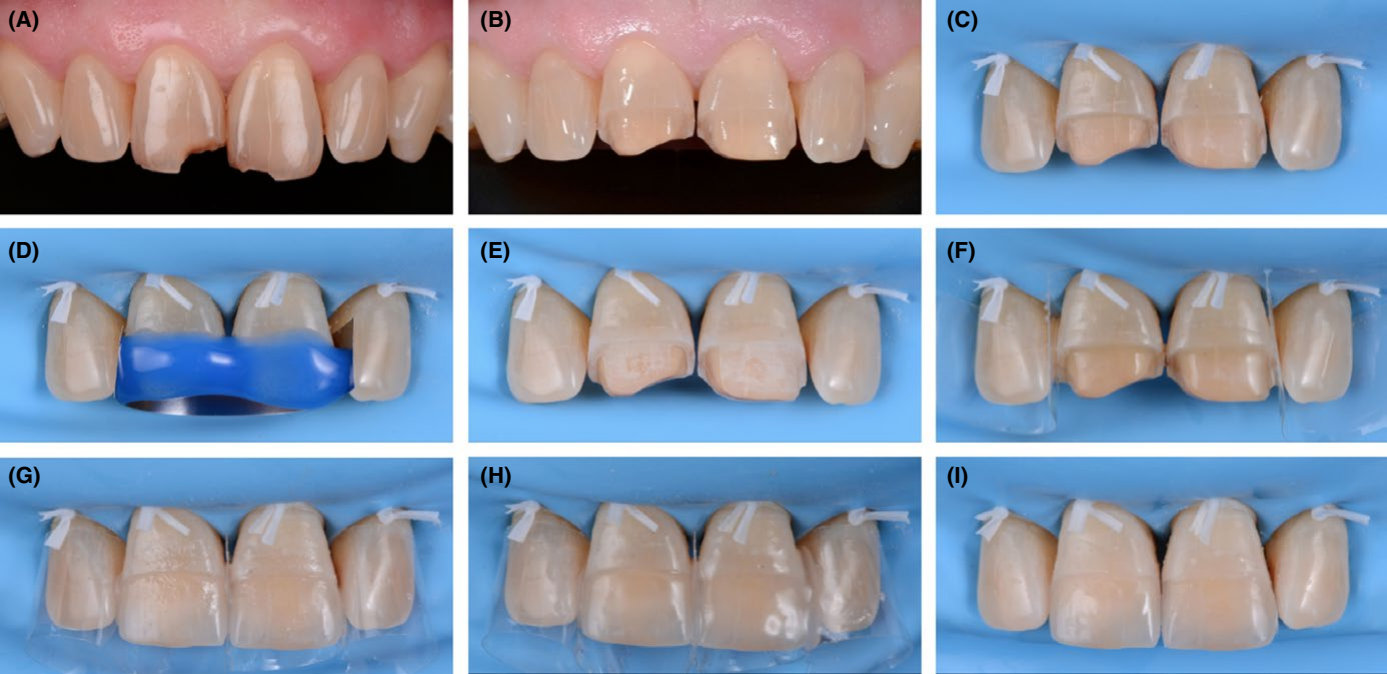
A, Initial situation (canine‐to‐canine view with contraster). B, Dental tissues after preparation. C, Isolation of the teeth using a rubber dam. D, Application of the etching agent. E, Rinsing with water and then drying. F, Application of the bonding agent. G, Application of partial veneers to the preparations. H, Photopolymerization using a glycerin film. I, Elimination of excess and polishing

The veneers were assembled with an adhesive resin cement (Variolink^®^Esthetic LC, Ivoclar‐Vivadent^®^) and gently applied to the teeth. Once the cement excess was removed with a brush, initial photopolymerization occurred, and a film of glycerin was applied at the limits of the preparation to protect the final photopolymerization of the bonding seal from exposure to oxygen. All the limits were thoroughly polished using polishing burs (Figure 6).

## 5 OUTCOME AND FOLLOW‐UP

**Figure 7:**
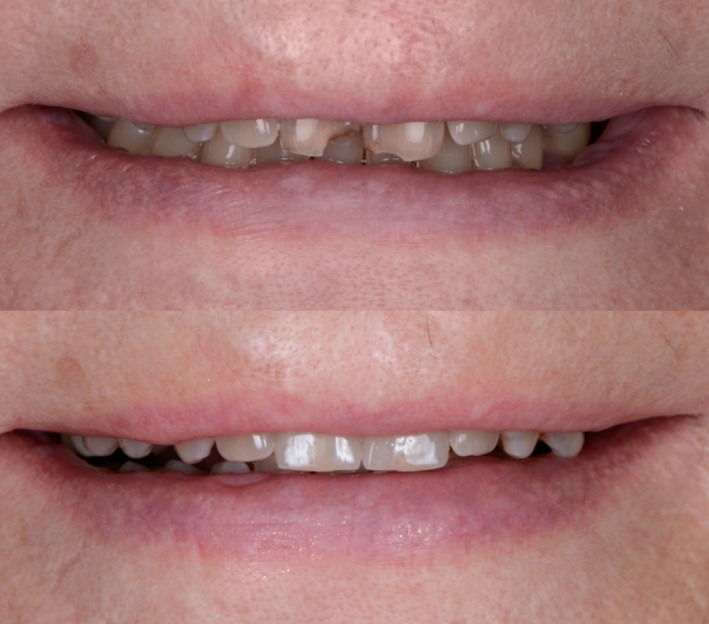
Before and After: in the final smile, the esthetics and function are restored, and the patient could pronounce phonemes without difficulty

In the presented clinical case, a 78‐year‐old patient with fractures of the two maxillary incisors was treated with partial ceramic veneers. Both teeth were restored with a natural appearance. The shape of the restorations (an incisal embrasure space arrangement where it increases in size and volume progressively distal from the central incisors) corresponded to the patient’s esthetic expectations while ensuring a balance between the incisive edge and lower lip between the dry and moist parts to allow for good allocution and phonemes^7^. The patient confirmed her satisfaction with the smile enhancement (Figure 7) after the procedure and during the one‐year follow‐up consultation.

Partial ceramic veneers correspond to all modern concepts including tissue economy, functional restoration, and high esthetics.

This technique seems an ideal method to restore a beautiful smile to patients with low smile lines, such as elderly people, as the limits of the restoration should not visible when the patient smiles.

## 6 DISCUSSION

The therapeutic decision is a key for the treatment of any case and it is motivated by many factors.

Tissue economy must always be considered in all therapeutic decision in every clinical case. Minimally invasive treatments, such as partial veneers, should therefore be considered first as they allow the preservation of the dental tissue while still providing highly satisfactory esthetic results. Furthermore, the possibility of maintaining maximal residual enamel after removing a mobile chip despite the cracked appearance is a reasonable choice because the dentin‐enamel junction can prevent any crack propagation in the dentin tissue.^8^ When the DEJ is intact, as in the presented case, it is advisable to opt for a minimally calibrated preparation to maximize tissue economy and for an optimal bonding.

While the practitioner choice should be guided by a therapeutic gradient with a systematic concern for saving residual tissue, partial ceramic veneer restoration seems a suitable alternative to direct composite restorations in the anterior area when reconstructing a limited defect^9^. As the biggest downside of this technique is the junction between the veneer and the enamel that may be visible, it seems especially adapted for treatment of the elderly who have been reported to present a low smile line hence preventing this border from being visible. Indeed, aging causes the smile to vertically narrow and to transversely widen while other studies suggest that muscles’ ability to form a smile also decreases with age.^10^

While the proportion of elderly citizens in “modern” populations is increasing, so is their likelihood of dental injury from fall. The work presented here describes the procedure for treating this kind of fractures and the relevance of using bonded partial restoration for the treatment of elderly patients, and more generally of patients of all ages with low smile lines.

## AUTHORSHIP

RC: is the main author of the manuscript and has led the treatment step by step. YA: contributed to the clinical case planning and the redaction of the manuscript by his expertise in the field of bonded ceramic veneer and esthetic dentistry. VP: contributed to the clinical case planning and the redaction of the manuscript by her expertise in the field of gerodontology. M‐FB: contributed to the clinical case planning and the redaction of the manuscript by her expertise in the field of restorative dentistry and minimally invasive approach.

## CONFLICT OF INTEREST

None declared.
